# Pre-conception and prenatal factors influencing gestational weight gain: a prospective study in Tigray region, northern Ethiopia

**DOI:** 10.1186/s12884-021-04171-z

**Published:** 2021-10-26

**Authors:** Kebede Haile Misgina, Eline M. van der Beek, H. Marike Boezen, Afework Mulugeta Bezabih, Henk Groen

**Affiliations:** 1 Department of Public Health, University of Aksum, College of Health Sciences, Axum, Ethiopia; 2 Department of Epidemiology, University Medical Center Groningen, University of Groningen, Groningen, The Netherlands; 3 Department of Paediatrics, University Medical Center Groningen, University of Groningen, Groningen, The Netherlands; 4 Department of Nutrition, University of Mekelle, Mekelle, Ethiopia

**Keywords:** Gestational weight gain, Socioeconomic factors, Body mass index, Ethiopia

## Abstract

**Background:**

In low-income countries, the high prevalence of pre-pregnancy undernutrition remains a challenge for the future health of women and their offspring. On top of good nutrition, adequate gestational weight gain has been recognized as an essential prerequisite for optimal maternal and child health outcomes. However, good-quality data on factors influencing gestational weight gain is lacking. Therefore, this study was aimed to prospectively identify pre-conception and prenatal factors influencing gestational weight gain in Ethiopia.

**Methods:**

A population based prospective study was undertaken between February 2018 and January 2019 in the Tigray region, northern Ethiopia. Firstly, the weight of non-pregnant women of reproductive age living in the study area was measured between August and October 2017. Subsequently, eligible pregnant women identified during the study period were included consecutively and followed until birth. Data were collected through an interviewer-administered questionnaire and anthropometric measurements complemented with secondary data. Gestational weight gain, i.e., the difference between 32 to 36 weeks of gestation and pre-pregnancy weights, was classified as per the Institute of Medicine (IOM) guideline. Linear, spline, and logistic regression models were used to estimate the influence of pre-conception and prenatal factors on gestational weight gain.

**Results:**

The mean gestational weight gain (standard deviation[SD]) was 10.6 (2.3) kg. Overall, 64.0% (95% CI 60.9, 67.1) of the women did not achieve adequate weight gain. Factors associated with higher gestational weight gain were higher women empowerment (B 0.60, 95% CI 0.06, 1.14), dietary diversity (B 0.39, 95% CI 0.03, 0.76), pre-pregnancy body mass index (B 0.13, 95% CI 0.05, 0.22), and haemoglobin (B 0.54, 95% CI 0.45, 0.64). Additionally, adequate prenatal care (B 0.58, 95% CI 0.28, 0.88) was associated with higher gestational weight gain.

**Conclusions:**

Adequate gestational weight gain was not achieved by most women in the study area, primarily not by those who were underweight before pregnancy. Interventions that advance women’s empowerment, dietary quality, pre-pregnancy nutritional status, and prenatal care utilization may improve gestational weight gain and contribute to optimizing maternal and child health outcomes.

**Supplementary Information:**

The online version contains supplementary material available at 10.1186/s12884-021-04171-z.

## Background

Weight gain during pregnancy, which is attributed to several physiologic changes, is normal. The physiologic changes include an increase in uterus’s size to accommodate the growing fetus, amniotic fluid, and placenta. Additionally, breasts enlarge, and blood and interstitial fluid volume rise. These changes contribute to increased weight, as do increased cellular water and maternal reserves [1, 2]. Adequate gestational weight gain, i.e., weight gain within the range recommended by the 2009 U.S. Institute of Medicine (IOM) guideline, is essential to gratify the metabolic demands of the women and their unborn children. Thereby, it could contribute to optimizing maternal and neonatal outcomes [2]. Examining the effects of weight gain across a range of pre-pregnancy body mass indices (BMI), the IOM guideline recommends that a weight gain of 12.5–18.0 kg for underweight, 11.5–16.0 kg for normal weight, 7.0–11.0 kg for overweight, and 5.0–9.0 kg for obese women. Inadequate weight gain or weight gain below the lower cut-offs has been shown to cause adverse maternal [3] and birth outcomes [3–10]. The adverse birth outcomes include preterm birth and low birth weight. Such adverse birth outcomes increase the risk of child mortality. For survivors, the adverse birth outcomes have negative consequences extending from early childhood across the life course and subsequent generations [3, 8, 11].

In low-income settings, where maternal undernutrition is common, inadequate gestational weight gain is also rampant [12–17]. According to a recent meta-analysis > 58% of women gained inadequate weight in sub-Saharan Africa including Ethiopia. Among underweight women, inadequate gestational weight gain ranged from 67.0 to 98.0% [12, 18]. Given the high prevalence of pre-pregnancy undernutrition [19], the omnipresent inadequate gestational weight gain remains a challenge for the future health of women as well as their offspring, perpetuating the cross-generational effects of chronic undernutrition in early life.

Different environmental and lifestyle factors may contribute to the actual gestational weight gain. These factors may include socioeconomic conditions [12], pregnancy plan, physical activity [2, 12], dietary practice [20], pre-pregnancy body mass index [18], distress [21, 22], and prenatal care [18]. However, the factors may vary with context [2, 12]. Most of the previous studies were from developed countries where adverse outcomes attributed to undernutrition are not prevalent. Besides, the limited numbers of studies in low-income settings were not or partially controlled for relevant confounders [4, 12]. Cognizant of this, factors influencing gestational weight gain are inconsistent and inconclusive. Furthermore, almost all studies in low-income settings relied on women’s recall of their pre-pregnancy weight or weight measured at prenatal care booking as a proxy for pre-pregnancy weight. In low-income settings, women hardly keep track of their body weight and often delay their prenatal care booking until the second trimester. As a result, the data are subjected to bias [12].

Therefore, there is a lack of good-quality data concerning gestational weight gain in low-income settings [12]. Such evidence is required to determine how adequate nutrition during the pre-pregnancy and pregnancy period can be used as a window of opportunity to improve maternal and child health. Such improvements could benefit the next generation in preventing the cross-generational perpetuation of chronic undernutrition. Thus, the present study was aimed to prospectively identify pre-conception and prenatal factors influencing gestational weight gain in Ethiopia.

## Methods

### Study design, setting, and population

The analyses for this study were performed on data from the **KI**lte-Awlaelo **T**igray **E**thiopia (KITE) cohort, a prospective cohort study in Kilite-Awlaelo Health and Demographic Surveillance Site (KA-HDSS) conducted between February 2018 and January 2019 [23]. KA-HDSS is located in the eastern zone of the Tigray region, northern Ethiopia. The site has 113,760 residents in ten rural and three urban kebeles (the smallest administrative units). Women of reproductive age account for 24% of the population. Within the surveillance site, about 4550 pregnancies are expected per year. Most of the population live in rural settings, and agriculture is the primary source of income.

Ethiopia has a three-tier health care system with health posts at the forefront of primary care. There is one health post in each kebele staffed by two to three Health Extension Workers (HEWs). Health posts provide promotional and preventive services under the umbrella of the ‘health extension package.’ The primary delivery modality of the package is through home-to-home visits. The health extension package consists of 16 components concerning family health services, disease prevention and control, and hygiene and environmental sanitation. The components include maternal and child health, family planning, nutrition, proper and safe waste disposal, and food hygiene and safety measures. Health Extension Workers train the households in their catchment area on health extension package and follow the progress of implementing the package after the training [24].

The sample size was calculated to achieve the objectives of the prospective study with respect to pregnancy outcomes in relation to nutritional status. The primary outcome was low birth weight, and the target was to be able to discriminate an estimated proportion of 24.6% low birth weight among women with Mid-Upper Arm Circumference (MUAC) ≥ 23.0 cm and a proportion of 32.6% among women with MUAC < 23.0 cm, as a cut-off to define undernutrition [25], with an alpha of 5% (2-sided), 80% power, and a 10% drop-out rate. The total sample size was calculated at 1100. With this sample size, differences in continuous outcomes > 0.2 standard deviations (SD) could also be detected.

Firstly, the weight of non-pregnant women (*n* = 17,500) living in the study area was measured between August and October 2017 using a Seca scale to the nearest 100 g. Subsequently, eligible pregnant women were identified and included consecutively between February 2018 and January 2019. A community-based survey by Health Extension Workers through the “Women Development Army,” a network of health information workers reaching individual households around the health posts, was applied to identify the pregnant women. Also, antenatal records and the KA-HDSS database were used to identify pregnant women. The criteria for inclusion were being married, being aged 18 or over, having weight measured before pregnancy, and having completed ≤20 weeks of gestation.

Identifying unmarried pregnant women is complicated by lack of registration that is also required to facilitate follow-up. In the study area, identification of women is based on identifying their respective households, which requires knowing the name of the household head (the husband). Therefore, only married women were included in the study, which was also an opportunity to collect husband information. As height continues to increase during the adolescence period, women aged < 18 were excluded to minimize bias in nutritional data.

### Measurements

An interviewer-administered questionnaire and anthropometric measurements were used to collect the data. Additionally, secondary data were extracted from the KA-HDSS database and antenatal records. The questionnaire was adapted from the literature [18, 25–29] and was pretested on a convenience sample of 55 pregnant women in Tahtay-Maichew, the central zone of the Tigray region. Qualified Health Extension Workers collected the data. The detailed descriptions of the measurements at different time points are provided below.

### At inclusion (at ≤20 weeks of gestation)

Age, residence (urban or rural), religion (Orthodox, Catholic, Muslim or others), educational status (no formal education, primary education, or secondary and above education), occupation (farmer, housewife or others), household size, and economic status were extracted from the KA-DHSS database. The database is updated every six months except for the economic status. For socioeconomic proxy indicator variables, adjustments were made at inclusion when there was a change since the last update. Then, economic status was determined by generating wealth index quintiles designating the lowest to the highest economic status from the proxy indicators. The socioeconomic proxy indicator variables included housing characteristics, access to improved water and sanitation facilities, and ownership of household assets, land, and livestock [30].

Access to improved drinking water sources refers to access to piped water on-premises, public taps or standpipes, tube wells or boreholes, protected dug wells, protected springs, and/or rainwater collection. Similarly, access to an improved sanitation facility was defined as access to an unshared toilet facility, pit latrine with a slab, ventilated improved pit latrine, or flush toilet [31]. Besides access, the time needed to fetch improved drinking water from the nearest source was collected at inclusion. Then, the self-reported time was dichotomized at a cut-off point of 30 min, with the time needed not exceeding 30 min indicating better service [31].

Moreover, access to the health facility was measured at inclusion by asking ‘How long does it take to go to the nearby health facility and back home?’. To assess the implementation of the health extension package, data were collected at inclusion by checking if the women’s respective households were certified as model households or not. Model household is a proxy for implementing the health extension package described above. For a household to be certified as a model, the requirement is receiving short-term training on the health extension package and implementing the package after the training [24]. Additionally, a short form of the International Physical Activity Questionnaire (IPAQ) was used to measure physical activity at inclusion, and the data were summarized as per the scoring protocol [29, 32]. Self-reported history of pre-pregnancy illnesses and perceived work burden were also collected at inclusion. Work burden was rated as easy, moderate, or difficult.

As part of the assessment of reproductive characteristics, the number of previous pregnancies, parity, history of abortion, and history of stillbirth, were extracted from the KA-DHSS database. Other reproductive factors like age at first marriage, age at first birth, a previous inter-birth interval in months, and history of preterm birth, delivery by C-section, and severe perinatal hemorrhage were collected by interview at inclusion. Based on this information, a history of adverse pregnancy outcomes was defined as having experienced one or more of the following: abortion, stillbirth, preterm birth, severe perinatal hemorrhage, or delivery by C-section.

Women were also asked to report on the four-item HITS (Hurt, Insult, Threaten, and Scream) scale at inclusion to assess intimate partner violence. Each question was scored from 1 to 5, and a total score > 10 was used as suggestive of violence [33]. Similarly, women’s empowerment was assessed by asking nine questions addressing three dimensions of empowerment: economic, socio-familial, and legal. The economic empowerment asked the relative income to husband, control over men’s income, control over women’s income, and decision-making on large household purchases. Likewise, the socio-familial empowerment was scored based on decision-making on family visits, decision-making on women’s health and attitude towards domestic violence. In contrast, the legal empowerment assessed women’s legal entitlements over land and house [34–36]. Then, coding each as 1 or 0, the scores were totaled as women empowerment scores (0 to 9). Furthermore, the question “At the time you became pregnant, did you wanted to get pregnant then, wanted later, or did not want at all?” was asked at inclusion to assess the index pregnancy plan. Accordingly, if the intention was not to get pregnant then, the pregnancy was considered unplanned.

Food and dietary characteristics, including the number of meals per day, frequency of dietary intake (vegetables, fruits, animals-source food, alcohol, and coffee), fasting, agrobiodiversity, harvest volume, dietary diversity, and food security, were assessed at inclusion. In assessing fasting, women were asked, ‘Do you fast?’. If you fast, ‘Which one: the regular weekly fast, the long fast times, or both?’. The weekly fast includes fasting every Wednesday and Friday almost throughout the year. The longer fasting periods include the 40-days Christmas fast, the 55-days of Lenten, the 14-days Apostles fast, and the14-days Dormition fast*.* Women were regarded as ‘fasting’ if they fasted both the weekly and the long fasting times.

Agrobiodiversity was captured by querying women about a list of food crops and livestock products their households produced in the past year. Then, by counting the number of product groups consisting cereals, roots, and tubers; legumes and nuts; oilseeds; fruits; vegetables; dairy; egg; and meat and poultry, a sum score of agrobiodiversity ranging from 0 to 8 was obtained [37]. Additionally, the amount of produce of each crop, expressed in quintiles was asked, and total harvest volume was calculated by adding the amounts reported for all crops.

A 24-h dietary diversity score was obtained by asking women if they consumed a list of food groups with ‘yes’ or ‘no’ response options. The sum yielded a dietary diversity score ranging from 0 to 10, with scores ≥5 indicating adequate diet diversity [27]. Moreover, to measure household food insecurity, women were asked how often nine specific food insecurity associated conditions, if any, happened in the previous month (0) not at all, 1) rarely, 2) sometimes, or 3) often) [28]. The answers were aggregated to a food insecurity score between 0 and 27. If the responses to all occurrence questions were no or if the affirmative response was only to “did you worry that your household would not have enough food” in a rare frequency of occurrence, households were classified as food secure [28].

Partner support was rated by the five-item Turner Support Scale with each item scored from 0 to 3 [38], and a sum score < 10 was defined as low. Support from significant other social sources was also rated using Oslo-3 Social Support Scale, and scoring ≤8 was considered low [39]. Both were summed up as a total support score. Distress was obtained using the ten-item Edinburgh Postnatal Depression Scale [40], the seven-item anxiety subscale of the Hospital Anxiety and Depression Scale [41], and the four-item Perceived Stress Scale [42]. The depression and anxiety scales were rated from 0 to 3, while the stress scale was scored from 0 to 4. Summing the standardized depression, anxiety, and stress scores, a total distress score was obtained. Additionally, a cut-off point ≥13 as suggestive of high depressive symptoms [40] and ≥ 8 for high symptoms of anxiety and stress were used. To indicate the level of distress, the presence of high symptoms in one, two, or all of the three domains, i.e., anxiety, depression, or stress, were considered.

Anthropometric measures; weight to the nearest 100 g as measured before pregnancy, and height to the nearest 0.1 cm were collected at inclusion using a Seca scale and height-measuring board. Likewise, MUAC to the nearest 0.1 cm was measured using MUAC-measuring tape. All measurements were taken twice and averaged. Pre-pregnancy BMI (pre-pregnancy weight (kg)/[height (m)]^2^) was categorized as underweight (BMI < 18.5), normal weight (BMI = 18.5 to 24.9), or overweight (BMI ≥ 25.0), and so was BMI calculated from weight and height measured at inclusion. MUAC below 21.0 cm was defined as undernutrition [43].

### In the third trimester (32 to 36 weeks)

At this stage, data on self-reported stressful life events that occurred in the past year, attendance of prenatal care, illness during pregnancy, and pregnancy complications were obtained [44]. Prenatal care was defined as adequate plus (five or more visits), adequate (four visits), or intermediate (two or three visits) for women who began prenatal care at ≤16 weeks of gestation. Prenatal care was defined as inadequate if started at >16 weeks or was received only once [45].

Moreover, weight and MUAC were measured as they were measured at inclusion. Also, data on human immunodeficiency virus (HIV) infection, urine analysis, rhesus factor, stool examination, venereal diseases, hepatitis B infection, haemoglobin, and other illnesses were extracted from antenatal records when available. Based on the measures at prenatal care booking, haemoglobin < 11 g/dL was defined as anaemia.

### Statistical analysis

Data were entered into Epi-Data 3.1 and analyzed with STATA (Version 11, Stata Corporation, College Station, Texas, USA). Proportions and means (SD) or medians (interquartile range [IQRs]) were used to describe the characteristics of the study population. Gestational weight gain, obtained by subtracting pre-pregnancy weight from weight measured between 32 to 36 weeks of gestation, was classified based on the 2009 IOM guideline. As excessive weight gain was rare, weight gain was re-categorized into inadequate or adequate. Student’s t-test or Mann Whitney U-test, as appropriate, was used to comparing the mean of continuous variables between women with adequate and inadequate weight gain. To compare the distribution of categorical variables by adequacy of weight gain, a Chi-squared test was used.

The assumption of linear association between gestational weight gain and the independent variables was preliminarily tested with ANOVA comparing mean weight gain by categories of each independent variable. If this test suggested non-linearity, spline regression was applied (Stata adjust-rcspline package), and each independent variable was partitioned into two continuous variables, below and above the knot value (K), using the mkspline command [46]. The coefficient B_2_ for the second spline variable represents the change in the effect of the variable above K as compared to below K. The effect of a unit increase in the value of the variable above K can be obtained as B_2_ − B_1_, where B_1_ is the coefficient of the lower partition. The knot value resulting in the best-fitted linear spline regression model, as apparent by the lowest root mean square of errors, was determined by checking the different values of the respective independent variable around a knot value estimated by viewing the linear spline regression curves. Finally, the two variables with their intercepts were regressed against gestational weight gain. If the coefficient for the second spline variable was statistically significant, this was considered to indicate that the effect of values above the knot was significantly different from below the knot, and we concluded that the association was non-linear. When the linear spline regression fitted better than quadratic and cubic models, the two variables and the second intercept were included in the analysis.

The unadjusted association of the independent variables with gestational weight gain was assessed using univariable linear regression. Including all statistically significant independent variables (*P* < 0.05, tested two-sided) from the univariable analyses, a multivariable linear regression model was fitted to determine adjusted effects on gestational weight gain. The normality of residuals was assessed through the normal probability and quantile-quantile plots. Homogeneity of variance was checked using the Breusch-Pagan test. Moreover, the model specification and omitted variable bias were tested using the Stata linktest and ovtest commands. Also, multicollinearity was assessed using the variance inflation factor (vif).

Finally, the probability of achieving adequate gestational weight gain by optimizing the foremost factors was estimated based on a multivariable logistic regression model using marginal standardization [47]. All independent variables that were significantly associated with adequate gestational weight gain (*P* < 0.05, tested two-sided) in the univariable analyses were included in the subsequent multivariable logistic regression model. Residence and MUAC were correlated with other variables and were not included.

## Results

**Fig. 1:**
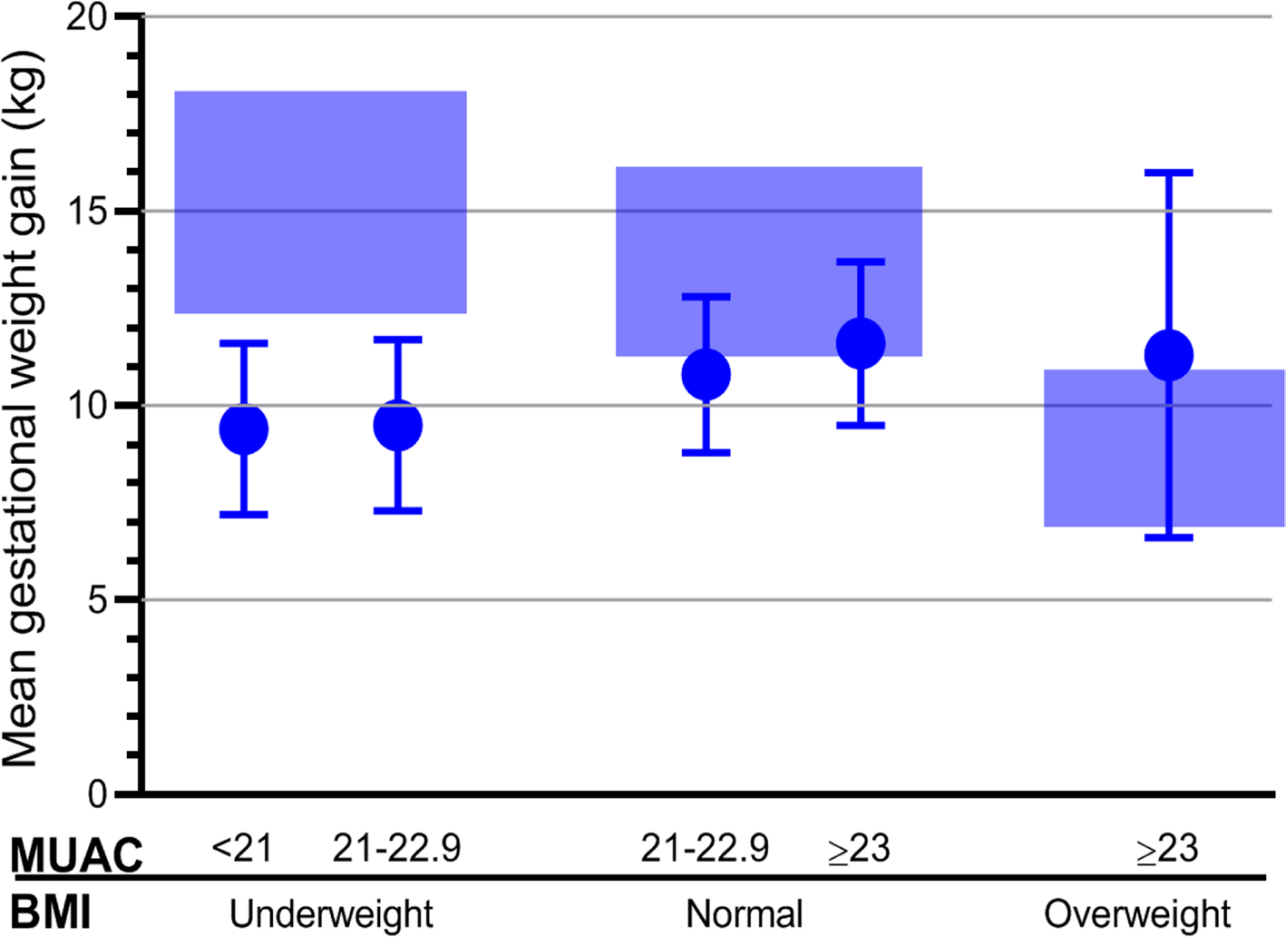
Average gestational weight gain by pre-pregnancy BMI (underweight: BMI < 18.5 (*n* = 335); Normal: BMI 18.5-25.0 (*n* = 594); overweight: BMI ≥ 25.0 (*n* = 5)) and by MUAC (< 21.0 cm versus 22.0-22.9 cm versus ≥23.0 cm) compared to the IOM recommended range of weight gain as shown by the blue shaded boxes, northern Ethiopia, 2018. Error bars indicate SD

Of the 991 pregnant women included at a mean (SD) of 14.8 (1.9) weeks of gestation, 934 were followed and their weight measured between 32 to 36 weeks of gestation (mean [SD] =33.9 [1.1] weeks). Table 1 summarizes the nutritional characteristics of the women by gestational weight gain. Before pregnancy, their average weight (SD) was 49.0 (6.6) kg with a respective BMI of 19.7 (2.0) kg/m^2^. The mean weight (SD) gained at inclusion was 2.4 (1.0) kg and 10.6 (2.3) kg at 32 to 36 weeks of gestation. Figure 1 provides weight gain by MUAC and BMI. Overall, 64.0% (95% CI 60.9, 67.1) of the women did not achieve adequate weight gain, with the prevalence being 91.6% among underweight women. On the other side of the gestational weight gain spectrum, 0.4% (95% CI 0.2, 1.1) of the women gained excessive weight.

**Table 1:** Summary of nutritional characteristics of women (*n* = 934) by gestational weight gain, northern Ethiopia, 2018

Characteristics	Gestational weight gain classification based on IOM recommendations						Total (*n* = 934)	
	Inadequate (***n*** = 598)		Adequate (***n*** = 332)		Excessive (***n*** = 4)			
	Mean (SD)	Range	Mean (SD)	Range	Mean (SD)	Range	Mean (SD)	Range
Height, cm	157.4 (0.1)	135.2 to 175.8	157.6 (0.1)	132.6 to 181.2	154.4 (0.0)	150.4 to 160.6	157.4 (0.1)	132.6 to 181.2
Pre-pregnancy weight, kg	47.5 (6.4)	31.8 to 68.9	51.7 (6.2)	33.3 to 72.9	57.0 (7.7)	47.9 to 66.1	49.0 (6.6)	31.8 to 72.9
Pre-pregnancy BMI, kg/m^2^	19.1 (1.9)	15.0 to 25.4	20.8 (1.7)	15.8 to 25.3	23.8 (2.2)	21.20 to 25.6	19.7 (2.0)	15.0 to 25.7
MUAC at inclusion, cm	22.0 (1.9)	17.5 to 29.3	23.6 (1.7)	18.1 to 28.3	26.6 (2.4)	23.8 to 29.6	22.6 (2.0)	17.5 to 29.3
Weight at inclusion, kg	49.6 (6.4)	34.2 to 71.5	54.6 (6.3)	36.6 to 75.7	60.0 (8.9)	49.5 to 70.9	51.5 (6.9)	34.2 to 75.7
Weight gain at inclusion, kg (*n* = 933)	2.15 (0.9)	0.6 to 5.3	2.9 (1.1)	1.2 to 6.4	3.1 (1.3)	1.6 to 4.8	2.4 (1.0)	0.6 to 6.4
BMI at inclusion, kg/m^2^	20.0 (1.9)	16.2 to 26.2	21.9 (1.7)	17.3 to 26.1	25.1 (2.6)	21.9 to 27.5	20.7 (2.1)	16.2 to 27.5
MUAC at 32 to 36 weeks, cm	22.2 (1.9)	17.8 to 29.3	23.8 (1.6)	18.3 to 28.3	26.8 (2.3)	24.20 to 29.8	22.8 (2.0)	17.8 to 29.8
Weight at 32 to 36 weeks, kg	56.8 (6.7)	39.7 to 78.8	64.6 (6.3)	44.9 to 88.3	73.3 (7.8)	64.3 to 83.1	59.6 (7.6)	39.7 to 88.3
Gestational weight gain, kg	9.3 (1.6)	5.0 to 14.8	13.0 (1.2)	8.7 to 17.0	16.4 (0.8)	15.3 to 17.0	10.6 (2.3)	5.0 to 17.0

Socioeconomic characteristics are shown in Table 2. At inclusion, the women’s average age (SD) was 29.3 (6.5), slightly more than a third (35%) were urban dwellers, 338 (36.2%) had no formal education, and a large majority (88.5%) were housewives or farmers. Significant differences between women with adequate and inadequate weight gain were seen in residence, family size, model household, physical activity, distress, and support from social sources (*P* < 0.05).

**Table 2:** Socioeconomic characteristics of women by gestational weight gain, northern Ethiopia, 2018

Characteristic	Total, ***n*** = 934	Gestational weight gain		***P***-value
		Adequate, ***n*** = 336	Inadequate, ***n*** = 598	
Age in year, mean (SD)	29.3 (6.5)	29.1 (6.4)	29.4 (6.6)	.376
Rural residence, n (%)	607 (65.0)	202 (60.1)	403 (67.4)	.031
Religion, n (%)				.764
Orthodox	922 (98.7)	331 (98.5)	591 (98.8)	
Others (Muslim and Catholic)	12 (1.3)	5 (1.5)	7 (1.2)	
Educational status of the woman, n (%)				.700
No formal education	338 (36.2)	120 (35.7)	218 (36.5)	
Primary education	307 (32.9)	109 (32.4)	198 (33.1)	
Secondary education or above	289 (30.9)	107 (31.9)	182 (30.4)	
Occupation of woman, n (%)				.158^a^
Farmer	506 (54.1)	168 (50.0)	338 (56.5)	
Housewife	321 (34.4)	126 (37.5)	195 (32.6)	
Employed	85 (9.1)	39 (11.6)	46 (6.7)	
Others^b^	22 (2.4)	3 (0.9)	19 (3.2)	
Educational status of husband, n (%)				.495
No formal education	300 (32.1)	104 (31.0)	196 (32.8)	
Primary education	345 (36.9)	124 (36.9)	221 (37.0)	
Secondary education or above	289 (31.0)	108 (32.1)	181 (30.2)	
Occupation of husband, n (%)				.073
Farmer	484 (51.8)	157 (46.7)	327 (54.7)	
Employed	212 (22.7)	83 (24.7)	129 (21.6)	
Daily laborer	148 (15.9)	64 (19.1)	84 (14.0)	
Others^c^	90 (9.6)	32 (9.5)	58 (9.7)	
Household size, mean (SD)	4.5 (2.0)	4.6 (2.1)	4.3 (1.9)	.042
Model household, n (%)	229 (24.5)	101 (30.1)	128 (21.4)	.004
Access to health facility in minutes, median (IQR)	32.5 (25-75)	40 (25-80)	30 (20-70)	.070^d^
Access to improved source of water, n (%)	835 (89.4)	302 (89.9)	533 (89.1)	.805
Time needed to fetch water not exceed 30 min, n (%)	745 (79.8)	266 (79.2)	479 (80.1)	.734
Access to improved sanitation facility, n (%)	132 (14.1)	46 (13.7)	86 (14.4)	.847
Household own < 0.5 ha farmland, n (%)	635 (68.0)	232 (69.0)	403 (67.4)	.654
Wealth index, n (%)				.483
Poorest	189 (20.2)	67 (20.0)	122 (20.4)	
Second poor	185 (19.8)	70 (20.8)	115 (19.2)	
Middle	190 (20.3)	73 (21.7)	117 (19.6)	
Second rich	186 (20.0)	70 (20.8)	116 (19.4)	
Rich	184 (19.7)	56 (16.7)	128 (21.4)	
Physical activity, n (%)				<.001
Low	497 (53.2)	208 (61.9)	289 (48.3)	
Moderate	404 (43.3)	122 (36.3)	282 (47.2)	
High	33 (3.5)	6 (1.8)	27 (4.5)	
Perceived work burden, n (%)				
Easy	383 (41.0)	146 (43.4)	237 (39.6)	.407
Moderate	414 (44.3)	141 (42.0)	273 (45.7)	
Difficult	137 (14.7)	49 (14.6)	88 (14.7)	
Low support from partner and others, n (%)	72 (7.7)	17 (5.1)	55 (9.2)	<.001
At least one stressful life event, n (%)	343 (36.7)	104 (30.9)	239 (40.0)	.002
Distress, n (%)				.006
Not distressed at all	518 (55.5)	207 (61.6)	311 (52.0)	
Distressed in one domain	206 (22.1)	75 (22.3)	131 (21.9)	
Distressed in two domains	122 (13.0)	36 (10.7)	86 (14.4)	
Distressed in three domains	88 (9.4)	18 (5.4)	70 (11.7)	
History of pre-pregnancy illness, n (%)	128 (13.7)	41 (12.2)	87 (14.5)	.317

^a^Occupation was compared by collapsing categories into three by merging the last two, ^b^Student, unemployed or others, ^c^Drivers, students, unemployed, or others, and ^d^Mann-Whitney U-test

As shown in Table 3, the mean (SD) parity among the non-nulliparous women was 3.3 (2.1). With 379 (40.6%) of the index pregnancies being unplanned, 437 (46.8%) women did not have adequate prenatal care, and 210 (22.5%) had a history of illness during the pregnancy. As to the food and dietary characteristics, many women (38.7%) belonged to food-insecure households, and the diversity of their diet was inadequate (47.2%). Significant differences were observed between women with inadequate and adequate weight gain in women empowerment, intimate partner violence, prenatal care, haemoglobin, diet diversity, food insecurity, and agrobiodiversity (*P* < 0.05).

**Table 3:** Reproductive and obstetric conditions as well as food and dietary characteristics of the participating women by gestational weight gain, northern Ethiopia, 2018

Reproductive and obstetric conditions	Total, *n* = 934	Gestational weight gain		*P*-value
		Adequate,* n* = 336	Inadequate, *n* = 598	
Age at first marriage in year, median (IQR)	18 (17-20)	18 (17-20)	18 (17-20)	.131^a^
Nulliparous before the index pregnancy, n (%)	181 (19.4)	112 (18.7)	69 (20.5)	.559
Parity (*n* = 753), mean (SD)	3.3 (2.1)	3.9 (1.8)	4.1 (1.9)	.113
Age at first birth in year (*n* = 747), median (IQR)	19 (18-21)	19 (18-21)	19 (18-20)	.176^a^
Previous birth interval in months (*n* = 558), median (IQR)	38 (30-48)	39 (33-48)	37 (30-47)	.085^a^
History of adverse birth outcome, n (%)	187 (24.8)	65 (19.3)	122 (20.4)	.887
Ever used modern contraceptive, n (%)	725 (77.6)	262 (78.0)	463 (77.4)	.911
Women empowerment score, mean (SD)	5.6 (1.5)	5.9 (1.5)	5.4 (1.4)	<.001
Intimate partner violence score, mean (SD)	6.9 (3.0)	6.2 (2.6)	7.3 (3.1)	<.001
Unplanned index pregnancy, n (%)	379 (40.6)	122 (36.3)	257 (43.0)	.055
Adequacy of prenatal care utilization, n (%)				.006
Inadequate	385 (41.2)	114 (33.9)	271 (45.3)	
Intermediate	112 (12.0)	41 (12.2)	71 (11.9)	
Adequate	380 (40.7)	156 (46.4)	224 (37.5)	
Adequate plus	57 (6.1)	25 (7.5)	32 (5.3)	
Iron-folic-acid tablets taken in two weeks, mean (SD)	10.2 (3.6)	10.6 (3.2)	10.4 (3.2)	.218
Haemoglobin (*n* = 882), mean (SD)	11.9 (1.6)	12.9 (1.3)	11.4 (1.5)	<.001
Anaemic (*n* = 882), n (%)	271 (30.7)	24 (7.5)	247 (43.8)	<.001
Number of pregnancy complications, median (IQR)	1 (0-2)	1 (0-2)	1 (0-2)	.177^a^
Number of pregnancy complications, n (%)				.098
None	428 (45.8)	139 (41.4)	289 (48.3)	
One	335 (17.6)	67 (19.9)	97 (16.2)	
Two or more	342 (36.6)	130 (38.7)	212 (35.5)	
History of diarrhea during pregnancy, n (%)	45 (4.8)	20 (6.0)	25 (4.2)	.292
Negative rhesus factor, n (%)	23 (2.5)	9 (2.8)	14 (2.3)	.900
Urine analysis positive result, n (%)	174 (18.6)	48 (14.3)	126 (21.1)	.014
Stool examination positive result, n (%)	30 (3.2)	14 (4.2)	16 (2.7)	.295
History of illness during pregnancy^b^, n (%)	210 (22.5)	21 (6.3)	36 (6.0)	1.000
**Food and dietary characteristics**				
Harvest volume in quintals, median (IQR)	0.75 (0-6)	0 (0-6.1)	3 (0-6)	.217^a^
Agrobiodiversity score, median (IQR)	2 (0-4)	1 (0-4)	2 (0-4)	.027^a^
Food insecure, n (%)	361 (38.7)	93 (27.7)	268 (44.8)	<.001
Adequate dietary diversity score, n (%)	493 (52.8)	216 (64.3)	277 (46.3)	<.001
Number of meals ≥3 times per day, n (%)	886 (94.9)	325 (93.8)	561 (96.7)	.075
Fruits intake ≥3 times per week, n (%)	53 (5.7)	21 (6.3)	32 (5.4)	.673
Vegetables intake ≥3 times per week, n (%)	86 (9.2)	34 (10.1)	52 (8.7)	.546
Animal-source food intake ≥3 times per week, n (%)	230 (24.6)	83 (24.7)	147 (24.6)	.967
Fasting, n (%)	650 (69.6)	209 (62.2)	441 (73.7)	<.001
Alcohol intake ≥1 times per week, n (%)	221 (23.7)	75 (22.3)	146 (24.4)	.521
Coffee intake ≥1 times per day, n (%)	738 (79.0)	266 (79.2)	472 (78.9)	.999

^a^Mann-Whitney U-test, and ^b^includes diarrheal diseases, malaria, sexually transmitted infections including HIV (human immunodeficiency virus), hepatitis and others

The results of multivariable linear regression analysis are depicted in Table 4. Excluding women with missing data, 882 were included in the final analysis. Women excluded from the final analysis did not differ from the remainder in all aspects except in residence and occupation. The difference in occupation was no longer significant after stratifying by residence (Additional file 1).

**Table 4:** Univariable and multivariable linear regression analysis of pre-conception and prenatal factors influencing gestational weight gain (*n* = 882), northern Ethiopia, 2018

Characteristics	Effect on gestational weight gain in kg (95% CI)			
	Unadjusted	*P*-value	Adjusted^**b**^	*P*-value
**Model 1**				
Women empowerment score below 4	−0.53 (−1.09, 0.02)	.060	− 0.48 (− 1.00, 0.04)	.069
Women empowerment score above 4	0.96 (0.39, 1.53)	.001	0.60 (0.06, 1.14)	**.028**
Dietary diversity score below 5	−0.20 (− 0.52, 0.11)	.208	− 0.32 (− 0.62, − 0.03)	**.030**
Dietary diversity score above 5	0.40 (0.01, 0.80)	.047	0.39 (0.03, 0.76)	**.035**
Food insecurity score	−0.10 (− 0.13, − 0.07)	<.001	− 0.01(− 0.05, 0.02)	.438
Number of meals per day^a^	2.39 (0.67, 4.13)	.007	1.16 (− 0.57, 2.88)	.189
Fruits intake (times per month)^a^	0.82 (0.39, 1.24)	<.001	0.24 (−0.28, 0.77)	.364
Alcohol intake (times per month)^a^	−0.71 (−1.18, − 0.23)	.004	−0.27 (− 0.73, 0.19)	.256
Fasting, yes	−0.59 (− 0.91, − 0.26)	<.001	− 0.13 (− 0.43, 0.16)	.379
Perceived work burden				
Easy	Reference	–	Reference	–
Moderate	−0.43 (− 0.75, − 0.11)	.008	− 0.26 (− 0.57, 0.05)	.097
Difficult	− 0.36 (− 0.79, 0.07)	.100	0.09 (− 0.37, 0.55)	.700
Physical activity				
Low	Reference	–	Reference	–
Moderate	−0.66 (− 0.96, − 0.36)	<.001	− 0.28 (− 0.60, 0.04)	.085
High	− 0.76 (−1.51, − 0.01)	.046	− 0.24 (− 1.00, 0.51)	.529
Total distress score	− 0.17 (− 0.23, − 0.12)	<.001	−0.003 (− 0.08, 0.07)	.916
Total support score	0.11 (0.07, 0.15)	<.001	0.02 (−0.03, 0.07)	.384
Intimate partner violence score	−0.12 (− 0.16, − 0.07)	<.001	−0.02 (− 0.07, 0.04)	.559
Pre-pregnancy BMI in kg/m^2^	0.43 (0.35, 0.51)	<.001	0.13 (0.05, 0.22)	**.003**
Husband occupation				
Farmer	Reference	–	Reference	**–**
Employed	0.39 (0.04, 0.74)	.028	−0.17 (−0.69, 0.36)	.533
Daily laborer	0.52 (0.09, 0.96)	.019	0.24 (− 0.27, 0.74)	.353
Others	0.03 (−0.49, 0.54)	.915	−0.27 (− 0.88, 0.34)	.383
Household size	−0.08 (− 0.16, − 0.01)	.032	− 0.003 (− 0.09, 0.08)	.932
Model household, yes	0.56 (0.22, 0.90)	.001	0.06 (− 0.26, 0.38)	.701
Agrobiodiversity score^a^	−0.49 (− 0.94, − 0.03)	.036	0.04 (− 0.76, 0.84)	.924
Number of stressful life events	− 0.27 (− 0.42, − 0.11)	.001	− 0.10 (− 0.24, 0.05)	.184
Adequacy of prenatal care utilization				
Inadequate	Reference	–	Reference	–
Intermediate	0.21 (−0.27, 0.70)	.387	0.07 (−0.35, 0.49)	.743
Adequate	0.80 (0.48, 1.12)	<.001	0.58 (0.28, 0.88)	**<.001**
Adequate plus	0.87 (0.22, 1.51)	.009	0.28 (−0.29, 0.86)	.334
Haemoglobin in g/dL	0.73 (0.65, 0.81)	<.001	0.54 (0.45, 0.64)	**<.001**
**Model 2**				
Quartiles of pre-pregnancy BMI				
First quartile (≤ 18.31)	Reference	–	Reference	**–**
Second quartile (18.32-19.48)	0.67 (0.26, 1.07)	.001	0.13 (−0.27, 0.53)	.517
Third quartile (19.49-20.99)	1.95 (1.54, 2.35)	<.001	0.71 (0.24, 1.17)	**.003**
Fourth quartile (≥ 21.00)	2.28 (1.88, 2.67)	<.001	0.77 (0.27, 1.27)	**.003**

^a^log transformed, and ^b^regression coefficients and 95% confidence intervals are shown. Model 1 was additionally adjusted for gestational age at the time when weight was measured in the third trimester, and Model 2 was obtained by re-running model 1 after replacing pre-pregnancy BMI with quartiles

In the adjusted model, higher women empowerment, dietary diversity, pre-pregnancy BMI, and haemoglobin were associated with higher gestational weight gain. Additionally, adequate prenatal care was associated with higher gestational weight gain. Precisely, a women empowerment score above four (B 0.60, 95% CI 0.06, 1.14) was positively associated with weight gain. Also, a U-shaped association between dietary diversity and weight gain was observed. A dietary diversity score below five (B −0.32, 95% CI −0.62, −0.03) was inversely related to weight gain. In contrast, the association between dietary diversity score above five (B 0.39, 95% CI 0.03, 0.76) and weight gain was positive. Moreover, higher pre-pregnancy BMI (B 0.13, 95% CI 0.05, 0.22), higher haemoglobin (B 0.54, 95% CI 0.45, 0.64) and adequate prenatal care (B 0.58, 95% CI 0.28, 0.88) were linked with higher gestational weight gain. In total, the model explained 33.8% of the variation in weight gain.

As shown in Table 5, the results of marginal standardization show that optimizing women’s economic empowerment, pre-pregnancy body mass index, dietary diversity, and adequate prenatal care would result in 50.1, 51.6, 55.2, and 41.4% probability of gaining adequate gestational weight, respectively.

**Table 5:** Results of marginal standardization showing the probability of gaining adequate gestational weight by optimizing the foremost prenatal and pre-conception factors as apparent in linear regression

Characteristics	Probability, % (95% CI)
Economic empowerment score	
≤ 3^rd^ quartile	43.1 (37.5, 48.6)
4^th^ quartile	50.1 (45.6, 54.7)
Socioeconomic empowerment score	
≤ 3^rd^ quartile	46.7 (41.8, 51.6)
4^th^ quartile	46.9 (42.0, 51.7)
Experiencing intimate partner violence	
Yes	20.3 (14.7, 25.9)
No	26.6 (22.3, 30.9)
Dietary diversity score	
≤ 3^rd^ quartile	44.2 (39.8, 48.7)
4^th^ quartile	55.2 (45.3, 65.2)
Fasting for religious purposes	
Yes	32.2 (29.1, 35.3)
No	37.4 (32.9, 42.8)
Pre-pregnancy body mass index	
Underweight	17.7 (11.1, 24.2)
Normal weight	51.6 (47.8, 55.4)
Haemoglobin at prenatal care booking	
< 11 g/dL	8.9 (5.5, 12.2)
≥ 11 g/dL	28.5 (24.3, 32.6)
Adequate prenatal care utilization	
Yes	41.4 (37.3, 45.6)
No	36.5 (32.8, 40.2)
Illness during the index pregnancy	
Yes	30.1 (24.5, 35.6)
No	38.7 (35.3, 42.0)
Experiencing at least one stressful life event	
Yes	30.2 (25.9, 34.6)
No	34.3 (30.3, 38.2)

## Discussion

In the present study, most women did not achieve adequate weight gain, primarily by those underweight before pregnancy. Women are missing both the pre-conception and pregnancy windows of opportunity with respect to improving maternal nutrition and optimizing maternal and child health. Higher women empowerment, dietary diversity, pre-pregnancy BMI, haemoglobin, and prenatal care were associated with higher gestational weight gain. The scientific evidence provided in this study indicated how the windows of opportunity could be used to improve maternal nutrition that could contribute to optimizing maternal and child health outcomes.

Our results on the prevalence of inadequate weight gain (64%) accord with low-income countries [12, 13, 15, 17, 18], although slightly lower than in Harari, Ethiopia (69.3%) [18]. Indeed, our results indicate that at the first measurement during pregnancy, there is already a relevant increase in body weight compared to a pre-pregnancy weight. This could merely justify the marginal incongruity with the study in Harari.

A unit increase in pre-pregnancy BMI was associated with higher gestational weight gain, aligning with the literature [18, 48, 49]. After replacing pre-pregnancy BMI with quartiles, only women in the third and fourth quartiles gained significantly higher weight. The association between higher pre-pregnancy BMI quartiles and higher weight gain implies that better nutritional status in the pre-conception period contributes considerably to maternal nutrition during pregnancy. This finding reflects the importance of paying due attention to the pre-pregnancy window. The insignificant weight gain among women with a lower quartile of pre-pregnancy BMI may also suggest that interventions during pregnancy may be too late for underweight women. Together, the findings may, therefore, highlight the benefit of starting public health interventions in the pre-conception period with a higher priority to underweight women to improve maternal nutrition. The interventions may include screening for undernutrition and considering multi-micronutrient supplements. Identifying and managing health problems that jeopardize maternal nutrition and nutritional education on dietary practices that affect intake and absorption of nutrients may also be relevant. For instance, coffee intake immediately before and/or after meals and fasting may impact dietary intake and absorption.

The association observed between pre-pregnancy BMI, and gestational weight gain may also suggest that the effectiveness of interventions targeting maternal nutrition during pregnancy depends largely on pre-pregnancy maternal nutritional status. However, our multivariable analyses revealed that other characteristics such as women’s empowerment and diversity of maternal diet have an independent association with gestational weight gain.

In the present study, a unit increase in women empowerment score above four was associated with a substantial increase in gestational weight gain. We are not aware of any published research that assessed the influence of women’s empowerment on weight gain, although some focused on related factors. Women empowerment reflects access to resources and decision-making power over resources and their own lives in and outside their households. Therefore, higher empowerment may imply better access to food, which can translate to improved dietary quality and maternal nutrition [35, 50–52]. Hence, the finding indicates the importance of taking evidence-driven actions to empower women.

Our data also showed a U-shaped association between dietary diversity and gestational weight gain. Though no previous study showed such a U-shaped association, some studies in fact linked adequate dietary diversity and sufficient weight gain or the reverse [20, 53–55]. The inverse association between diversity of diet scores below five and weight gain may indicate that an increase in dietary diversity score may only be beneficial if it increases substantially, crossing the threshold score of 5 food groups. On the other hand, the positive association between dietary diversity scores above five and gestational weight gain may have been due to the nutrient adequacy containing higher energy and micronutrient required during pregnancy to attain the recommended weight gain [56]. Therefore, the association between dietary diversity and weight gain may provide an interesting point of attention in any actions that should focus on promoting dietary diversity of women regardless of other socio-economic circumstances.

Additionally, the positive association found between haemoglobin and gestational weight gain, as shown in Malawi [15], may also reflect the importance of promoting maternal dietary quality. Similarly, it may imply the need for strengthening the interventions already practiced like iron and folic acid supplementation and deworming. Conversely, as pregnancy advances, the increase in plasma volume may be followed by haemodilution leading to lower haemoglobin concentration which is opposite in direction to weight change at least until 35 to 38 weeks of gestation [57, 58].

In the present study, however, haemoglobin was measured at prenatal care booking, and the majority of the women gained inadequate weight. The time when haemoglobin was measured and the inadequate weight gain signify that the change in plasma volume and their pre-pregnancy nutritional status was low. Thus, the haemoglobin measures might be close to the early pregnancy state and indicate the nutritional condition of the women in early pregnancy. Moreover, a positive association was observed between adequate prenatal care and weight gain during pregnancy, as reported previously [59]. The association found between prenatal care and weight gain during pregnancy may also suggest that promoting access to and adequate use of prenatal care may be essential to improve maternal nutritional status.

Some major strengths of the study include using a population-based prospective design, assessment of pre-conception nutritional status, and detailed data collection to evaluate several nutrition-sensitive factors. The limitations of the study includes, for instance, that pre-pregnancy weight might have changed between the time of measurement and immediately before conception in some women. However, considering the weight measured before pregnancy is still better than the weight at the first prenatal care booking, which could be as far as 20 weeks into the pregnancy. In addition, the seasonal influence in dietary diversity was not well addressed, and haemoglobin was not adjusted for altitude. Finally, some of the variables assessed in our study were sensitive by nature, and our study might not have been free of social desirability bias.

## Conclusions

Adequate gestational weight gain was not achieved by most women in the study area, primarily not by those who were underweight before pregnancy. Interventions that advance women’s empowerment, dietary quality, pre-pregnancy nutritional status, and prenatal care utilization may improve gestational weight gain and contribute to optimizing maternal and child health outcomes.

## Supplementary Information

**Additional file 1.** Baseline characteristics of women who were and were not included in the complete-follow-up sample and final analysis, northern Ethiopia 2018.

## Data Availability

The data that supports the findings of this study are available from the corresponding author on reasonable request.

## Abbreviations

BMI: Body mass index
CI: Confidence interval
HITS: Hurt, Insult, Threaten, and Scream
HIV: Human immunodeficiency virus
IPAQ: International Physical Activity Questionnaire
IQR: Interquartile range
IOM: Institute of Medicine
KA-HDSS: Kilite-Awlaelo Health and Demographic Surveillance Site
MUAC: Mid-Upper Arm Circumference
SD: Standard deviation
vif: Variance inflation factor
