# Supplementary material for: Pre-conception and prenatal factors influencing gestational weight gain: a prospective study in Tigray region, northern Ethiopia
Source: BMC Pregnancy Childbirth. 2021 Oct 26;21:718. doi: 10.1186/s12884-021-04171-z (PMC8546955; doi:10.1186/s12884-021-04171-z)
Supplement: Supplementary file 1 — Additional file 1. Baseline characteristics of women who were and were not included in the complete-follow-up sample and final analysis, northern Ethiopia 2018. [file 12884_2021_4171_MOESM1_ESM.docx]

**Additional file 1** Baseline characteristics of women who were and were not included in the complete-follow-up sample and final analysis, northern Ethiopia 2018

| **Characteristics** | **Included, *n*=882** | **Not included, *n*=109** | ***P*-value** |
| --- | --- | --- | --- |
| Age at inclusion, mean (SD) | 29.5 (6.3) | 29.3 (6.5) | .802 |
| Rural residence, n (%) | 554 (62.8) | 93 (85.3) | **.000** |
| Educational status of woman, n (%) |  |  |  |
| No formal education | 311 (35.6) | 51 (43.6) | .114 |
| Primary education | 287 (32.8) | 39 (33.3) |  |
| Secondary and above | 276 (31.6) | 27 (23.1) |  |
| Occupation of woman, n (%) |  |  | **.010** |
| Farmer | 464 (52.6) | 77 (70.6) |  |
| Housewife | 314 (35.6) | 23 (21.1) |  |
| Employed | 83 (9.4) | 8 (7.4) |  |
| Others^a^ | 21 (2.4) | 1 (0.9) |  |
| Occupation of woman (only for rural), n (%) |  |  | .924 |
| Farmer | 463 (83.6) | 77 (82.8) |  |
| Housewife | 61 (11.0) | 10 (10.8) |  |
| Others | 30 (5.4) | 6 (6.4) |  |
| Occupation of woman (only for urban), n (%) |  |  | .716 |
| Housewife | 253 (77.1) | 13 (81.2) |  |
| Others | 75 (22.9) | 3 (18.8) |  |
| Wealth index, n (%) |  |  | .882 |
| Poorest | 178 (20.2) | 20 (18.4) |  |
| Second poor | 175 (19.8) | 23 (21.1) |  |
| Middle | 181 (20.6) | 19 (17.4) |  |
| Second rich | 175 (19.8) | 25 (22.9) |  |
| Rich | 173 (19.6) | 22 (20.2) |  |
| Food insecurity score, median (IQR) | 0 (0-8) | 0 (0-8.5) | .063^b^ |
| Access to improved drinking water, n (%) | 790 (89.6) | 98 (89.9) | .913 |
| Time to fetch water within 30 minutes, n (%) | 700 (80.1) | 88 (75.2) | .269 |
| Access to improved sanitation facility, n (%) | 127 (14.4) | 8 (7.3) | .060 |
| Household size, mean (SD) | 4.5 (2.0) | 4.7 (2.1) | .175 |
| Parity, mean (SD) | 2.6 (2.3) | 2.9 (2.3) | .198 |
| Unplanned index pregnancy, n (%) | 357 (40.5) | 48 (44.0) | .477 |
| History of pre-pregnancy illness, n (%) | 122 (13.8) | 20 (18.3) | .204 |
| Women empowerment score, mean (SD) | 5.6 (1.5) | 5.8 (1.6) | .060 |
| Intimate partner violence score, mean (SD) | 6.9 (3.0) | 6.8 (3.2) | .763 |
| Total support score, mean (SD) | 21.3 (3.9) | 21.9 (3.4) | .111 |
| Number of stressful life events, median (IQR)^b^ | 0 (0-1) | 0 (0-1) | .684 |
| Distress score, mean (SD) | -0.0 (2.6) | 0.1 (2.4) | .763 |
| Dietary diversity score, mean (SD) | 4.6 (1.4) | 4.4 (1.4) | .249 |
| Maternal height in cm, mean (SD) | 157.5 (0.1) | 157.7 (0.1) | .730 |
| MUAC at inclusion in cm, mean (SD) | 22.6 (0.1) | 22.2 (0.2) | .055 |
| Pre-pregnancy BMI kg/m^2^, mean (SD) | 19.8 (2.0) | 19.4 (1.6) | .072 |
| BMI at inclusion kg/m^2^, mean (SD) | 20.7 (2.1) | 20.4 (1.6) | .114 |
| History of illness during pregnancy, n (%) | 203 (23.0) | 22 (20.2) | .500 |
| Gestational weight gain in kg, mean (SD) | 11.9 (1.6) | 11.6 (1.2) | .163 |

^a^Student, unemployed or others, and ^b^Mann-Whitney U-test
